# Supplementary figures and images for: Parallel evolution leading to impaired biofilm formation in invasive Salmonella strains
Source: PLoS Genet. 2019 Jun 24;15(6):e1008233. doi: 10.1371/journal.pgen.1008233 (PMC6611641; doi:10.1371/journal.pgen.1008233)

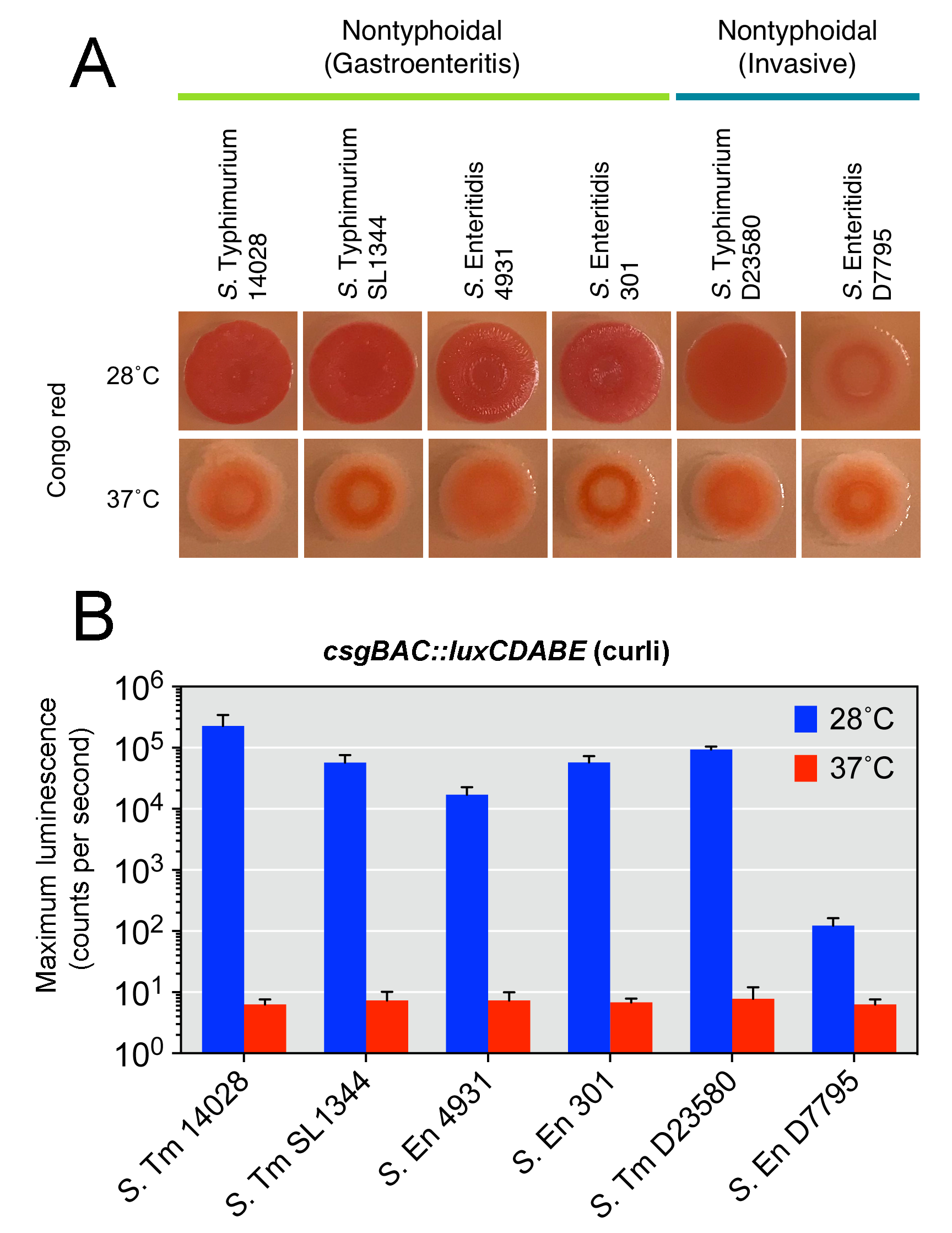

Supplement: S1 Fig — (A) Morphological comparison of colonies grown for five days at 28˚C or 37˚C on T agar supplemented with 40 μg mL-1 Congo red. (B) Maximum expression from a curli-specific reporter (csgBAC::luxCDABE) in each strain grown for 48 h at 28˚C or 37˚C. Luciferase expression was measured every 30 min during continuous culture. (TIF) [file pgen.1008233.s001.tif]

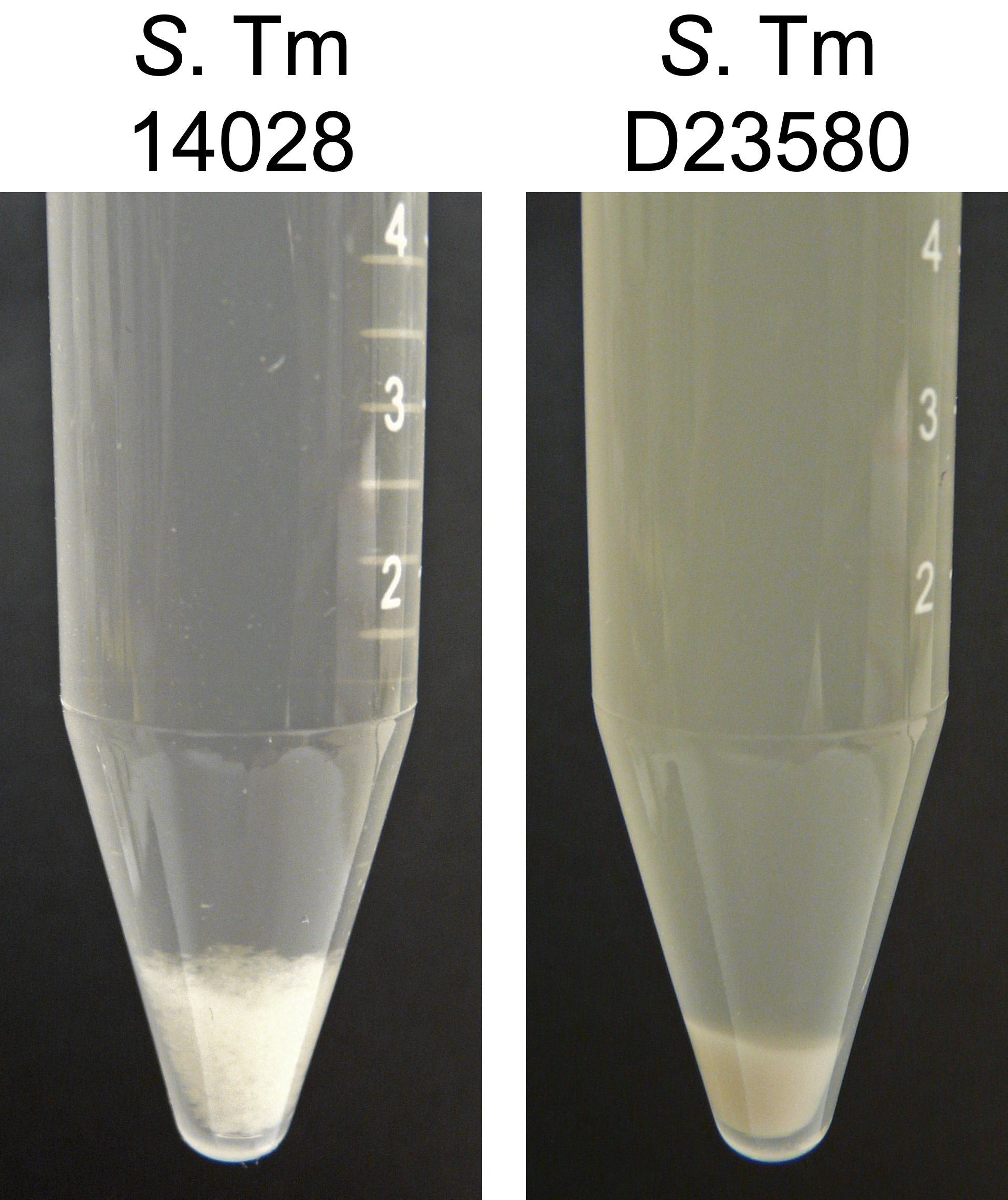

Supplement: S2 Fig — Multicellular aggregates and planktonic cells formed from liquid cultures of S. Typhimurium 14028 and S. Typhimurium D23580. Images match those presented in Fig 1, but have been enhanced to emphasize aggregates within the samples. Aggregates formed by S. Typhimurium D23580 cells appear structurally distinct from aggregates formed by S. Typhimurium 14028 cells. (TIF) [file pgen.1008233.s002.tif]

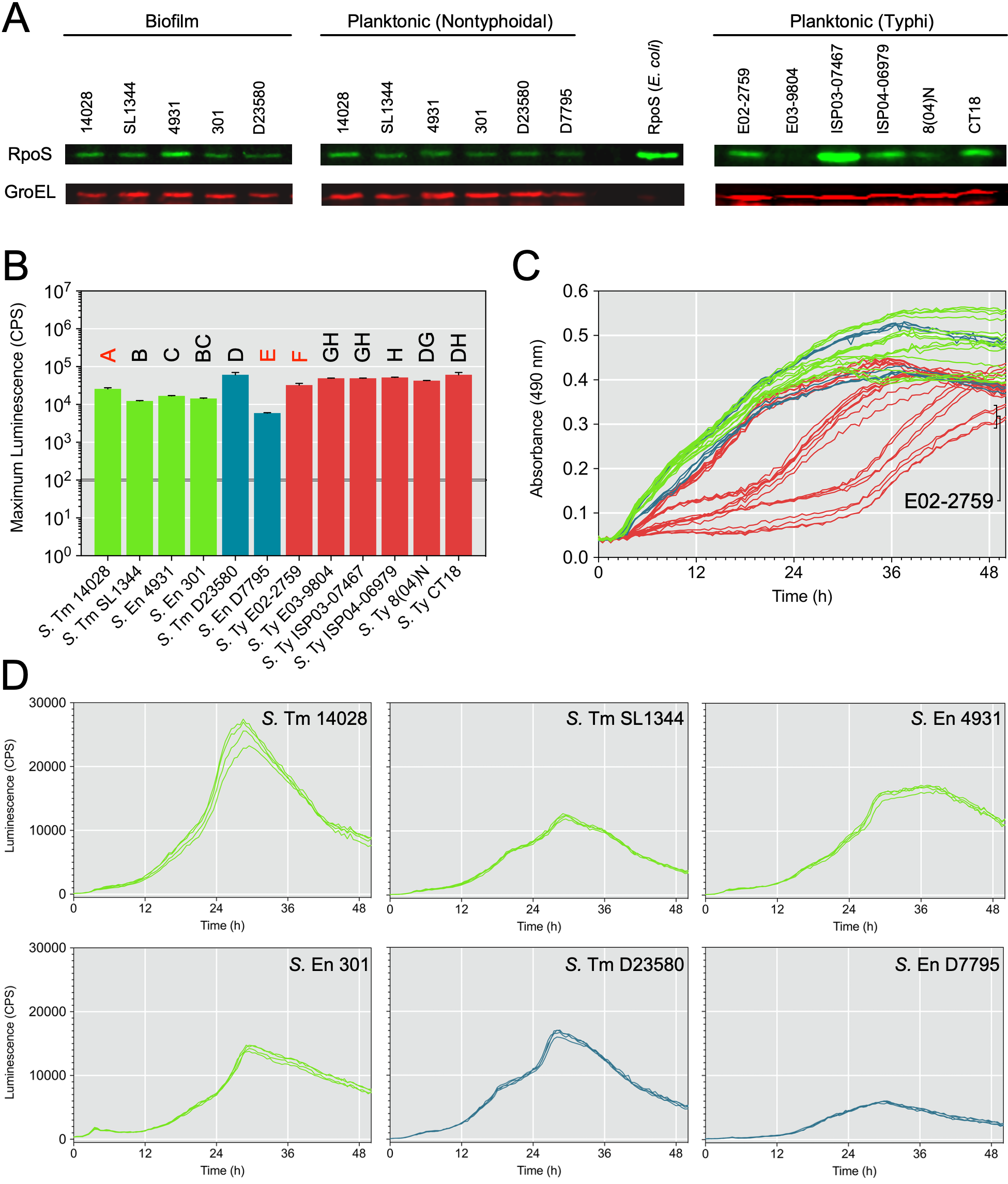

Supplement: S3 Fig — (A) Whole cell lysates were generated from multicellular aggregates and planktonic cells isolated from flask cultures of Salmonella strains after 24 hours of growth and probed for synthesis of RpoS. Lysates were normalized by total protein concentration. GroEL was used as a loading control to ensure that equal amounts of protein were loaded into each sample lane. (B) RpoS activity was evaluated by measuring luminescence from a synthetic, RpoS-dependent promoter-reporter construct expressed in each Salmonella strain during 48 hours of growth. Graphed values represent the maximum reporter activity recorded and is reported as counts per second (CPS). (C) Absorbance measurements represent strain growth in microaerophilic conditions in 96-well microtiter plates. Each curve represents one biological replicate; n = 4 per strain. (D) Time course of RpoS-dependent promoter activity in each indicated strain. (TIF) [file pgen.1008233.s003.tif]

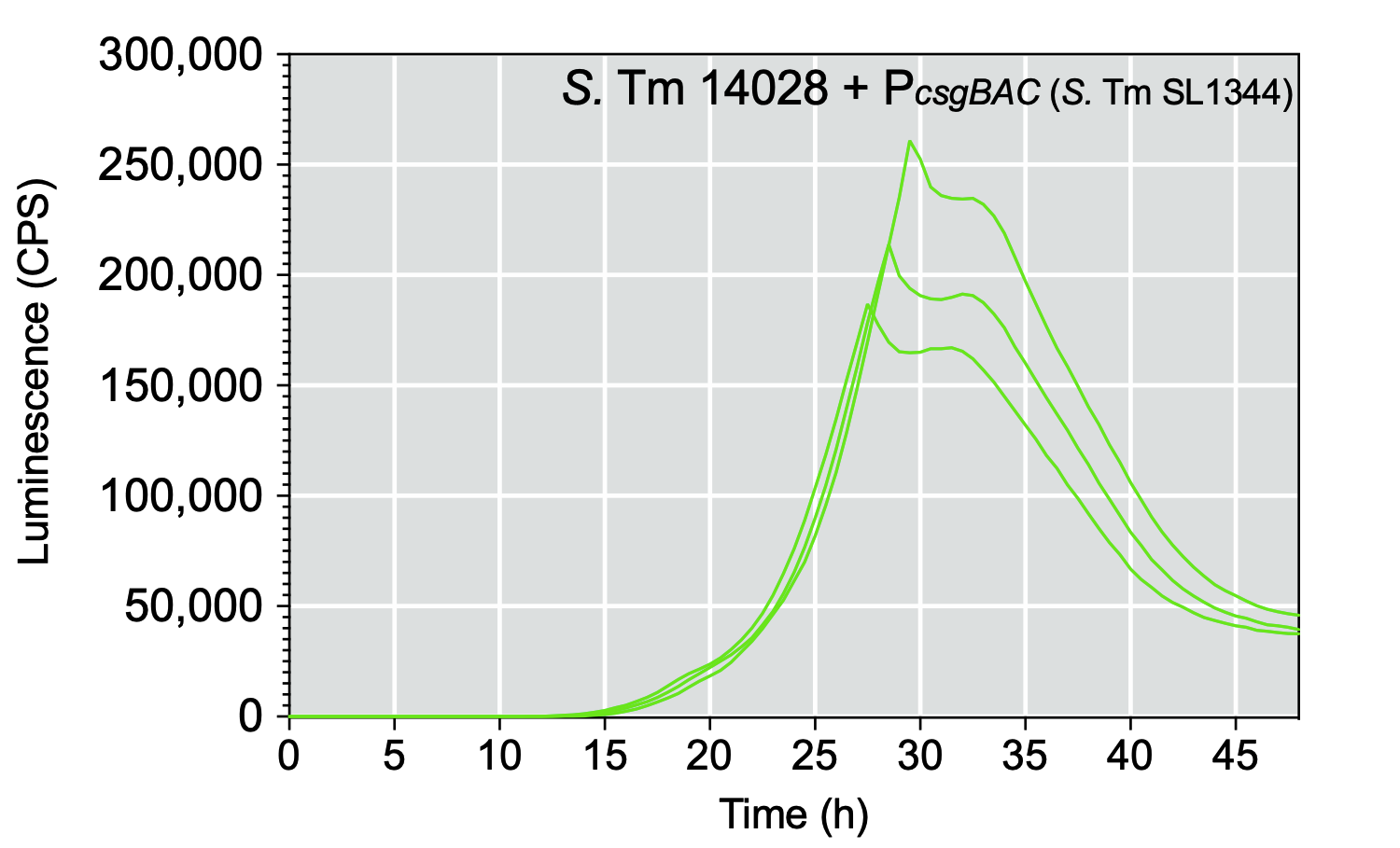

Supplement: S4 Fig — Each curve represents one biological replicate; n = 3. (TIF) [file pgen.1008233.s004.tif]

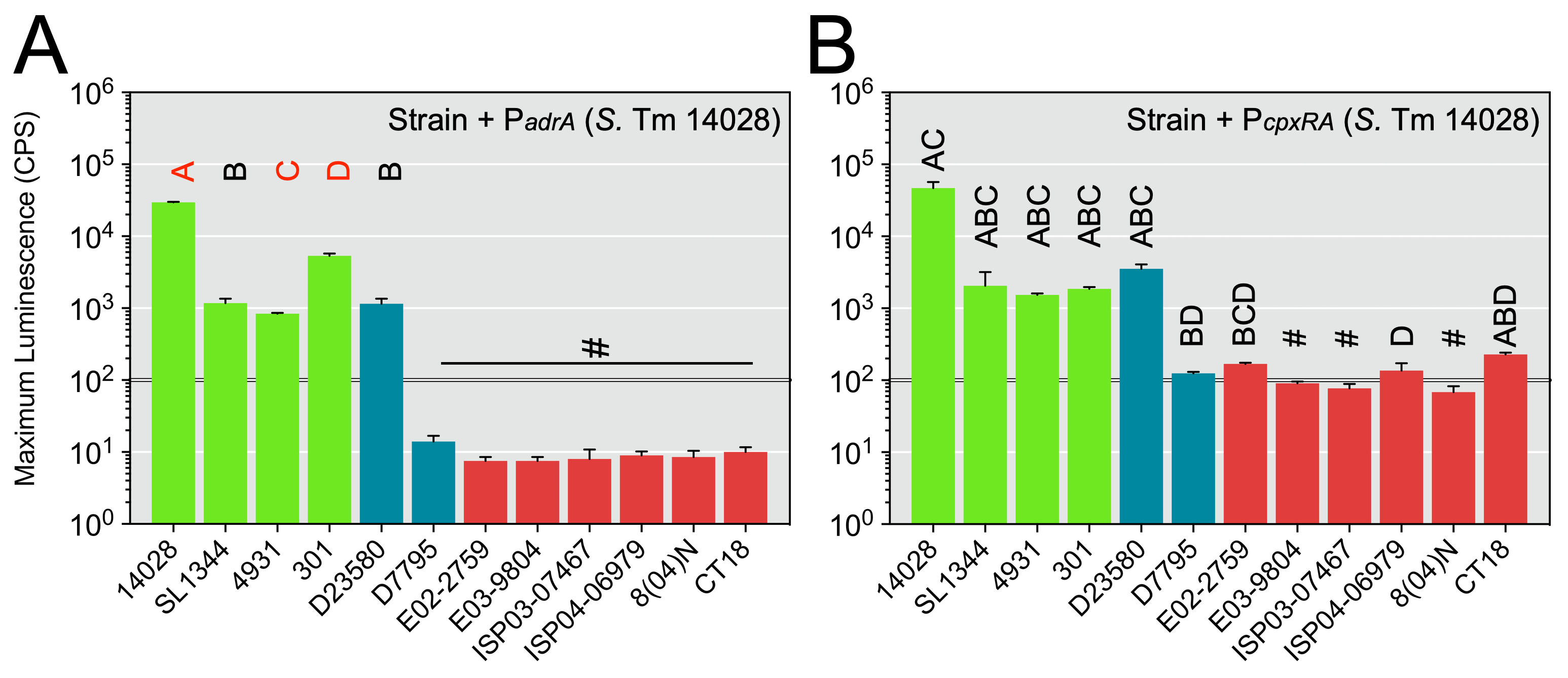

Supplement: S5 Fig — Promoter-reporter constructs derived from S. Typhimurium 14028 adrA and cpxRA promoter sequences were introduced into each of the strains. Letters above the bars indicate mean values that were statistically similar to (black font) or different from (red font) other mean values. #, values below the activity threshold as established in [24]. Each bar represents the mean value from 3–5 independent biological replicates and error bars represent the standard deviations. (TIF) [file pgen.1008233.s005.tif]

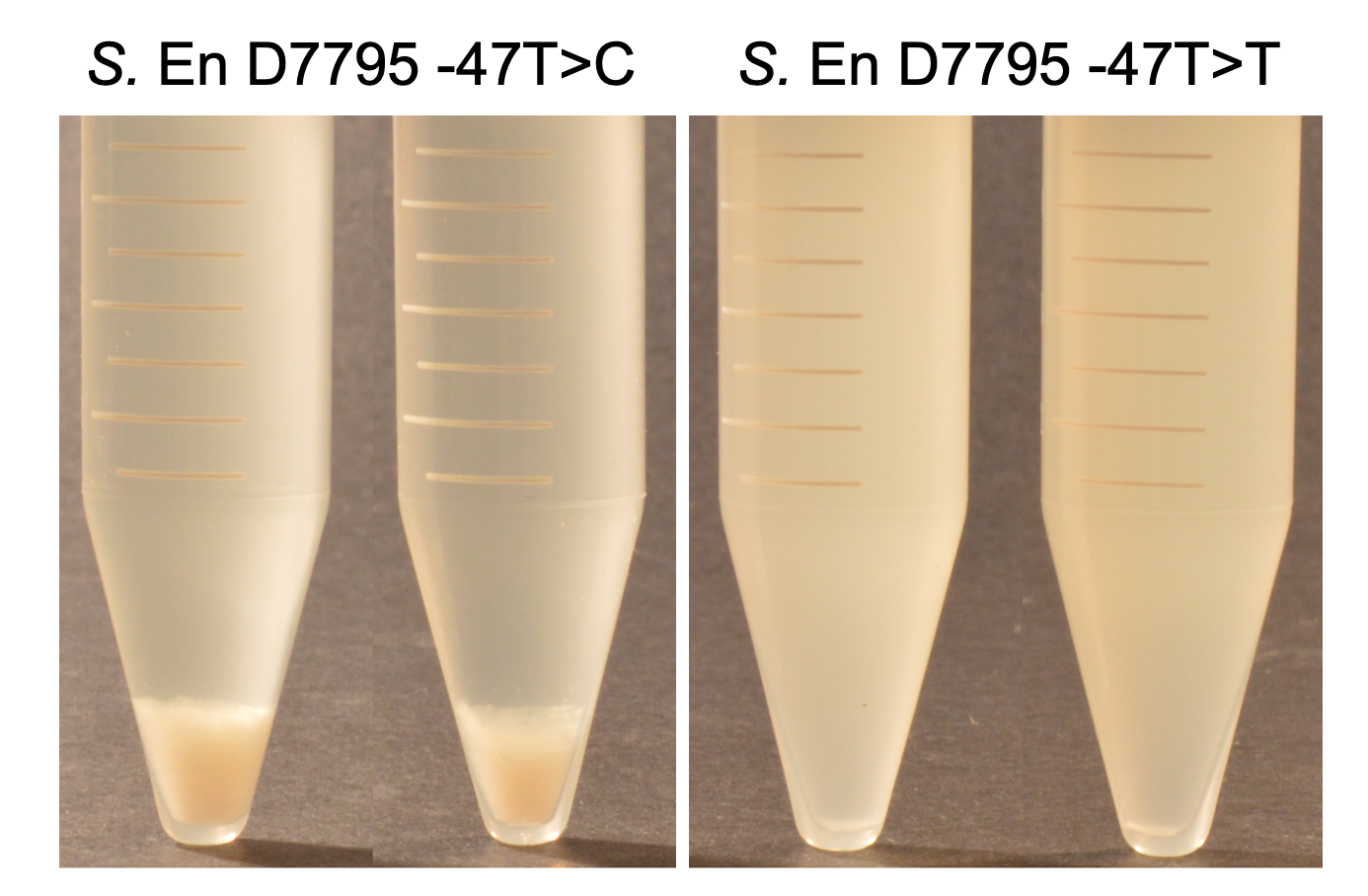

Supplement: S6 Fig — Clones that contain (-47T>T) or do not contain (-47T>C) the identified ‘T’ promoter SNP at position -47 were grown in flasks of 1% tryptone for 24 hours before being evaluated for the ability to form aggregates in liquid cultures. (TIF) [file pgen.1008233.s006.tif]

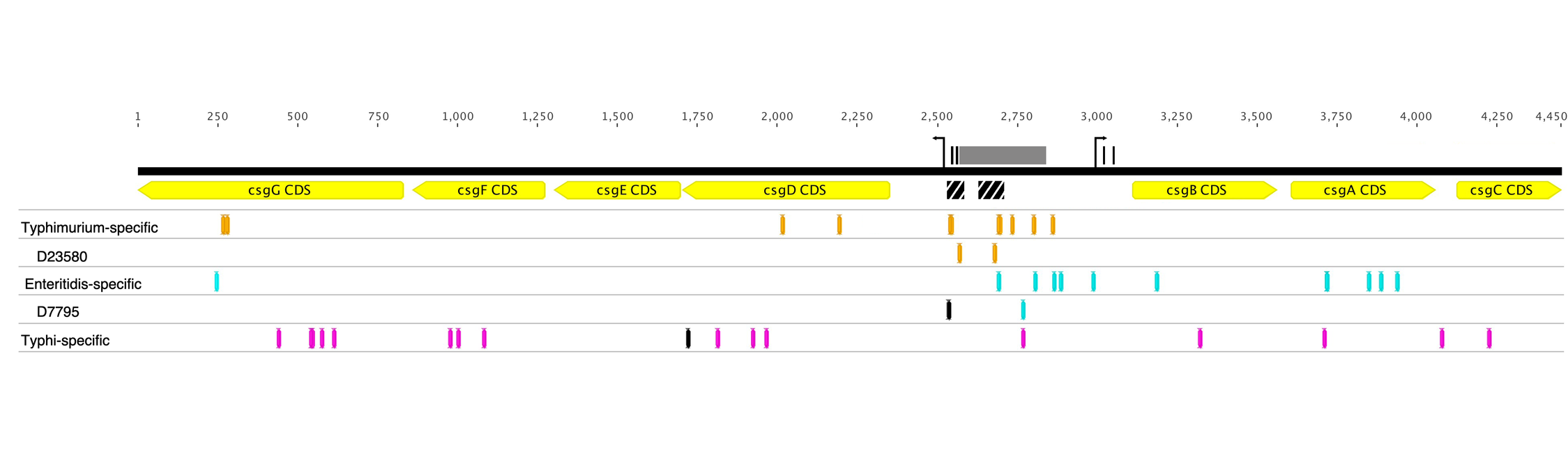

Supplement: S7 Fig — Simplified multiple sequence alignment of the csgDEFG and csgBAC operons highlighting serovar- and strain-specific single nucleotide polymorphisms representing both nonsynonymous and synonymous mutations: S. Typhimurium and D23580 (yellow), S. Enteritidis and D7795 (light blue), and S. Typhi (pink). Other highlighted changes (black) include the SNP in D7795 that inactivates csgD transcription, and the SNP in S. Typhi strains that introduces a premature stop codon in csgD, yielding a CsgD protein that is truncated by 8 amino acids. The long black bar represents the DNA region with nucleotide position numbers listed above and csg genes shown below as yellow-boxed arrows. Special sequence features involved in operon regulation are highlighted above and below the black bar: -35 and -10 promoter regions (black elbow arrows), H-NS binding region (grey box), CpxR-binding sites (black bars), and OmpR binding regions (hatched boxes). (TIF) [file pgen.1008233.s007.tif]

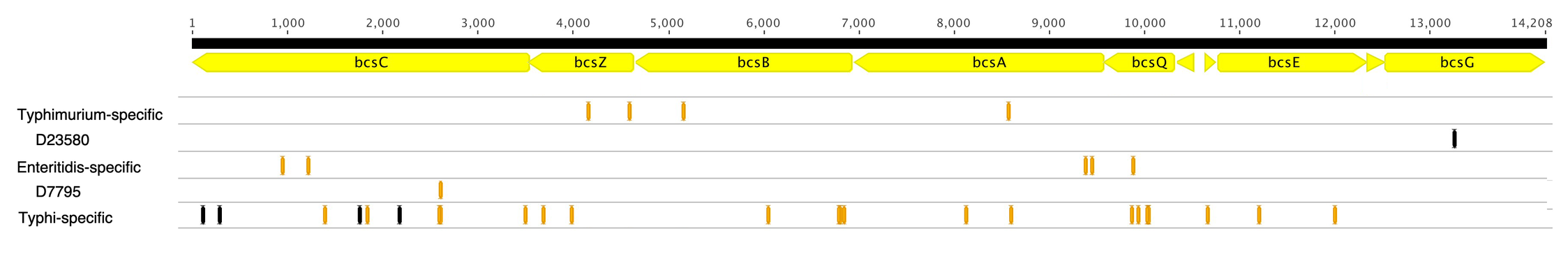

Supplement: S8 Fig — Simplified multiple sequence alignment of the bcsRQABZC and bcsEFG operons highlighting non-synonymous serovar- and strain-specific single nucleotide polymorphisms. Non-synonymous SNPs are shown in yellow; black bars indicate SNPs that yield premature stop codons (i.e. bcsG in D23580; four SNPs in bcsC in S. Typhi strains). The long black bar represents the DNA region with nucleotide position numbers listed above and bcs genes shown below as yellow-boxed arrows. bcsR, bcsF and a small hypothetical protein are shown without their names listed. (TIF) [file pgen.1008233.s008.tif]
