# Supplementary material for: Parallel evolution leading to impaired biofilm formation in invasive Salmonella strains
Source: PLoS Genet. 2019 Jun 24;15(6):e1008233. doi: 10.1371/journal.pgen.1008233 (PMC6611641; doi:10.1371/journal.pgen.1008233)
Supplement: S1 Table — (PDF) [file pgen.1008233.s009.pdf]

Table S1. Strains and plasmids used in this study.

**Strain or Plasmid**	**Genotype**	**Source or Reference**
Strains		
S. Typhimurium SL1344	Wild-type strain	ATCC (W Köster, U of S)
S. Enteritidis ATCC 4931	Wild-type strain	ATCC (D Korber, U of S)
S. Enteritidis 301 (Sal18)	Wild-type strain	W Köster [1]
S. Typhi E02-2759	Wild-type strain	G. Dougan [2]
S. Typhi E03-9804	Wild-type strain	G. Dougan [2]
S. Typhi ISP03-07467	Wild-type strain	G. Dougan [2]
S. Typhi ISP04-06979	Wild-type strain	G. Dougan [2]
S. Typhi 8(04)N	Wild-type strain	G. Dougan [2]
S. Typhi CT18	Wild-type strain	G. Dougan [3]
S. Enteritidis D7795	Wild-type strain	G. Dougan [4]
-47 T>C	P*csgD* from 14028	This study
-47 T>T	Native P*csgD*[a]	This study
S. Typhimurium D23580	Wild-type strain	G. Dougan [5]
-80 A>C	P*csgD* SNP from 14028	This study
-80 A>C, -189 A>G	P*csgD* SNPs from 14028	This study
S. Typhimurium 14028	Wild-type strain	ATCC
-47 C>T	P*csgD* from D7795	This study
-47 C>C	Native P*csgD*[a]	This study
bcsG$_{(D23580)}$	*bcsG* from D23580	This study
bcsG$_{(14028)}$	Native *bcsG*[a]	This study
csgD$_{(CT18)}$	*csgD* from CT18	This study
csgD$_{(14028)}$	Native *csgD*[a]	This study
Plasmids		
pCS26, pU220	Bacterial luciferase	[6]
pCS26-csgB	*csgBAC* promoter	[7]
pU220-csgD	*csgDEFG* promoter	[7]
pCS26-sig38H4	Synthetic, RpoS-responsive promoter	[7]
pCS26-adrA	*adrA* promoter	[7]
pCS26-cpxR	*cpxR* promoter	This study
pFLAG-CTC		Sigma-Aldrich
p3xFLAG	Modified to have 3xFLAG at C-terminus	This study
p3xFLAG/*csgD*^CT18	*csgD* from *S.* Typhi CT18	This study
p3xFLAG/*csgD*^14028	*csgD* from *S.* Typhimurium 14028	This study

[a] These strains were selected at the end of the genome engineering procedure, and possessed promoter sequences or alleles that were the same as the parent strain

SUPPLEMENTARY REFERENCES

1. Poppe C. *Salmonella* infections in the domestic fowl. Salmonella in domestic animals. Wallingford: CABI; 2000. pp. 107–132. doi:10.1079/9780851992617.0107
2. Holt KE, Parkhill J, Mazzoni CJ, Roumagnac P, Weill F-X, Goodhead I, et al. High-throughput sequencing provides insights into genome variation and evolution in *Salmonella* Typhi. Nature Genetics. 2008;40: 987–993. doi:10.1038/ng.195
3. Parkhill J, Dougan G, James KD, Thomson NR. Complete genome sequence of a multiple drug resistant *Salmonella enterica* serovar Typhi CT18. Nature. 2001;413: 848–852. doi:0.1038/35101607
4. Feasey NA, Hadfield J, Keddy KH, Dallman TJ, Jacobs J, Deng X, et al. Distinct *Salmonella* Enteritidis lineages associated with enterocolitis in high-income settings and invasive disease in low-income settings. Nature Genetics. 2016;48: 1211–1217. doi:10.1038/ng.3644
5. Kingsley RA, Msefula CL, Thomson NR, Kariuki S, Holt KE, Gordon MA, et al. Epidemic multiple drug resistant *Salmonella* Typhimurium causing invasive disease in sub-Saharan Africa have a distinct genotype. Genome Res. 2009;19: 2279–2287. doi:10.1101/gr.091017.109
6. Bjarnason J, Southward CM, Surette MG. Genomic profiling of iron-responsive genes in *Salmonella enterica* serovar typhimurium by high-throughput screening of a random promoter library. Journal of Bacteriology. American Society for Microbiology (ASM); 2003;185: 4973–4982. doi:10.1128/JB.185.16.4973-4982.2003
7. White AP, Gibson DL, Kim W, Kay WW, Surette MG. Thin Aggregative Fimbriae and Cellulose Enhance Long-Term Survival and Persistence of *Salmonella*. Journal of Bacteriology. 2006;188: 3219–3227. doi:10.1128/JB.188.9.3219-3227.2006
