# Supplementary material for: Parallel evolution leading to impaired biofilm formation in invasive Salmonella strains
Source: PLoS Genet. 2019 Jun 24;15(6):e1008233. doi: 10.1371/journal.pgen.1008233 (PMC6611641; doi:10.1371/journal.pgen.1008233)
Supplement: S2 Table — (PDF) [file pgen.1008233.s010.pdf]

S2 Table. Oligonucleotides used in this study.

Primer	Sequence (5′ - 3′)[a]	Purpose
cpxR1	GCCCTCGAGGTAACTTTGCGCATCGCTTG	To amplify the *cpxRA* promoter region from *Salmonella* genomic DNA
cpxR2	GCCGGATCCTTCATTGTTTACGTACCTCCG	
agfD1	GTGCTCGAGGGACTTCATTAAACATGATG	To amplify the *csg* intergenic region for cloning into luciferase reporter plasmids
agfD2	GCCGGATCCTGTTTTTCATGCTGTCAC	
agfD3-FWD	GTACCTGCAGGGACTTCATTAAACATGATG	To amplify the *csg* intergenic region from *Salmonella* genomic DNA for genome engineering
agfD4-REV	GTCAGGATCCTGTTTTTCATGCTGTCAC	
agfD5-FWD	GTACCTGCAGTGGTTAACAATCCGGG	To amplify the *csg* intergenic region and csgD ORF from *Salmonella* genomic DNA for genome engineering
agfD6-REV	GTCAGGATCCGGGGCGAATAGCCATA	
bcsG checkF	GATCGGATCCGGATTTCTCCAGTTCGGTAA	To amplify *bcsG* from *S.* Typhimurium D23580 for DNA sequencing and genome engineering
bcsG checkR	GATCCTGCAGTGGTATGGCTGAATGTACTG	
3xFLAG-linker A	TCGAGAATGTAGCAGATCTGGACTACAAAGACCATGACGGTGATTATAAAGATCATGACA	To construct the p3xFLAG vector from pFLAG-CTC
3xFLAG-linker B	TCGATGTCATGATCTTTATAATCACCGTCATGGTCTTTGTAGTCCAGATCTGCTACATTC	
csgD-ORF-start	GATCCTCGAGGTCCATAGTAGTCATGGTCA	To amplify the *csgD*^14028 and *csgD*^CT18-alleles for cloning into p3xFLAG
csgD-ORF-end	GATCGGATCCCGCCTGAGATTATCGTTTGC	
csgDORFstartPstI	GATCCTGCAGGTCCATAGTAGTCATGGTCA	To amplify the *csgD*-containing DNA region from *S.* Typhi CT18 for genome engineering
csgDreplaceREV	GATCGGATCCCGAAGACGACGGTTTTCTC	

[a] Nucleotide sequences corresponding to restriction enzyme sites are underlined.
